# Supplementary material for: Diet and chemical defenses of the Sonoran Desert toad
Source: PLoS One. 2025 Nov 10;20(11):e0335661. doi: 10.1371/journal.pone.0335661 (PMC12599970; doi:10.1371/journal.pone.0335661)
Supplement: S2 Table — (DOCX) [file pone.0335661.s002.docx]

**S2 Table**

***Incilius alvarius***

| **Toad Species** | **Sample Treatment** | **Number of Samples** | **Number of Toads** |
| --- | --- | --- | --- |
| *I. alvarius* | Sampled in one vial according to procedure outlined in Methods | 17 | 17 |
| *I. alvarius* | Sample split across two vials due to large size of parotoid gland | 6 | 3 |
| *I. alvarius* | Sampled into a second vial due to suspected contamination from blood spotting in the original vial as an accidental result of toxin extraction.  For one toad, only the second, clean, sample is available (original blood-contaminated sample was not available for analysis) | 5 | 3 |
| *I. alvarius* | Sample split across three vials due to large size of parotoid gland *and* blood spotting as an accidental result of toxin extraction. | 3 | 1 |
| *I. alvarius* | Sample split across two vials for the parotoid gland, and leg glands (one vial per leg). | 4 | 1 |
| *I. alvarius* | Sampled from the parotoid and leg gland (combined in one vial), dorsal skin and ventral skin (one vial each). | 3 | 1 |
|  | TOTAL | 39 | 26 |

**Other Toads**

| **Toad Species** | **Sample Treatment** | **Number of Samples** | **Number of Toads** |
| --- | --- | --- | --- |
| *A. punctatus* | Sampled once from the parotoid gland | 6 | 6 |
| *A. cognatus* | Sampled once from the parotoid gland | 3 | 3 |
