# Supplementary material for: Diet and chemical defenses of the Sonoran Desert toad
Source: PLoS One. 2025 Nov 10;20(11):e0335661. doi: 10.1371/journal.pone.0335661 (PMC12599970; doi:10.1371/journal.pone.0335661)
Supplement: S3 Table — (DOCX) [file pone.0335661.s003.docx]

**S3 Table**

| **Molecule**  **Composition**  *(adduct)* | ***Anaxyrus punctatus***  *(#present/*  *#toads measured)* | ***Anaxyrus cognatus***  *(#present/#toads measured)* | ***Incilius***  ***alvarius****  *(#present/#toads measured)* | **Reported presence for *Incilius alvarius* only** | **Reported presence for *A. punctatus* and *A. cognatus*** |
| --- | --- | --- | --- | --- | --- |
| 5-MeO-DMT (*-H*) | 1/6 | 1/3 | 26/26 | Ersparmer 1967; Uthaug 2019; Schwelm 2021 | First report** |
| Bufotenine  C12H16N2O  *(-H)* | 3/6 | 1/3 | 21/26 *17/26 from the first sample vial only* | Ersparmer 1967; Uthaug 2019; Schwelm 2021 | [(Cei et al., 1968)](https://paperpile.com/c/HgFXOn/1Zas) |
| 5-MeO-N-methyltryptamine  C12H16N2O  (-H) | 3/6 | 1/3 | 21/26 *17/26 from the first sample vial only* | Ersparmer 1967; Uthaug 2019; Schwelm 2021 | First report |
| MeO-tryptamine  C11H14N2O  (-H) | 1/6 | 2/3 | 1/26 | Schwelm 2021 | First report |
| 5-MeO-tryptophol  C11H13NO2  *(-OH)* | 0/6 | 0/3 | 1/26  *Found on ventrum and dorsum, not parotoid glands* | Uthaug 2019; Schwelm 2021 | None |
| Tryptophan  C11H12N2O2  *(-H)* | 3/6 | 1/3 | 3/26  *Found on ventrum, dorsum and second-round secretions only* | Ersparmer  1967; Schwelm 2021 | First report |

*Totals calculated across all 39 samples available for chemical analysis, including toads where secretions were split across two or three vials. We noted any cases where compounds were found only in dorsal or ventral glands, or only in the second sample vial.

** All *I. alvarius* secreted 5-MeO-DMT. We also documented small amounts of 5-MeO-DMT (<15% abundance) in the secretions of one *A. punctatus* and several *A. cognatus* in the positive-ion mode only. This observation is likely an error but may merit follow-up study to determine whether either of these species do produce 5-MeO-DMT in small amounts, as Sonoran Desert toads are currently the only animal confirmed to secrete the compound.
