# Supplementary figures and images for: Diet and chemical defenses of the Sonoran Desert toad
Source: PLoS One. 2025 Nov 10;20(11):e0335661. doi: 10.1371/journal.pone.0335661 (PMC12599970; doi:10.1371/journal.pone.0335661)

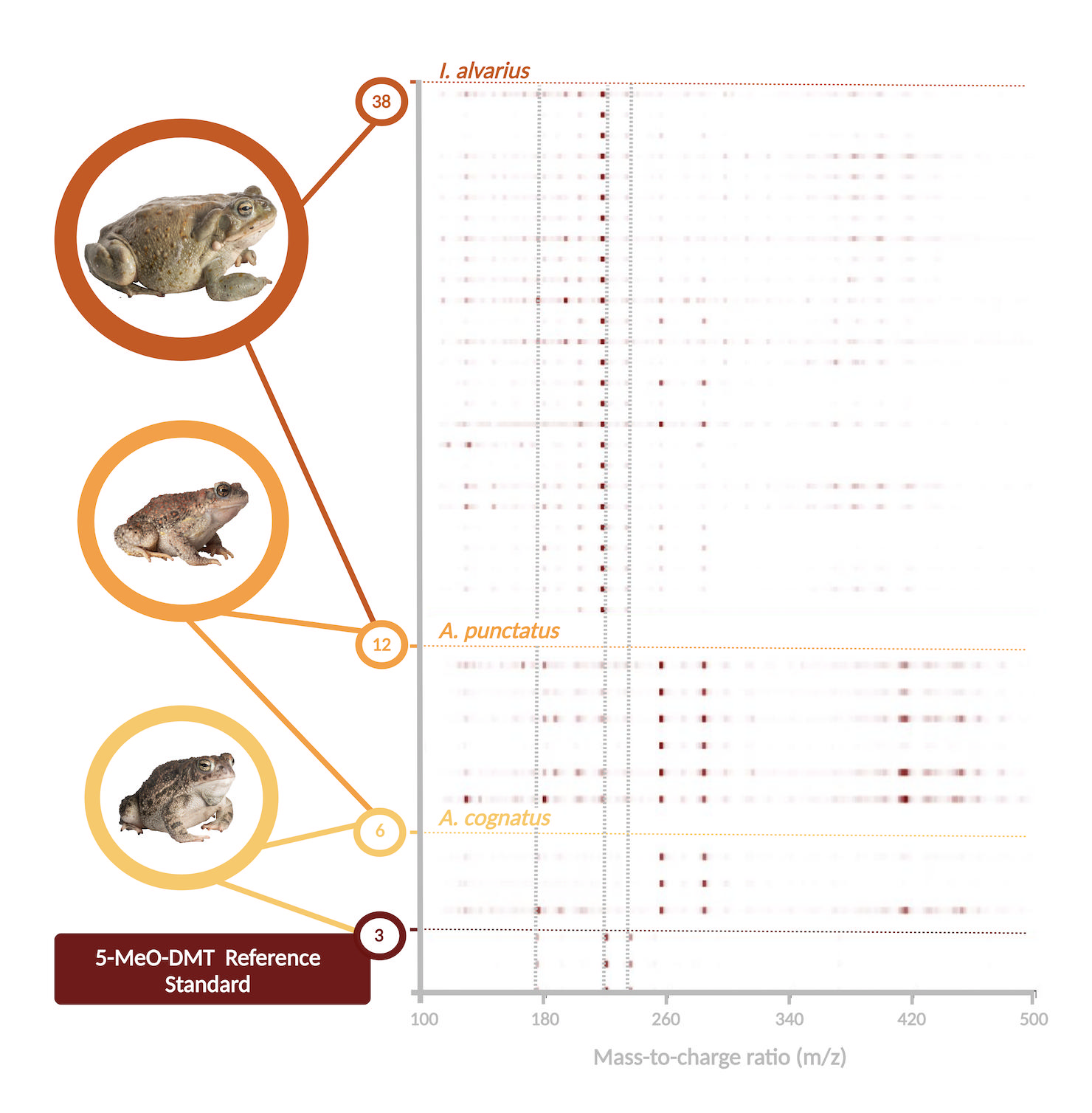

Supplement: S1 Fig — (TIF) [file pone.0335661.s005.tif]
