# Supplementary material for: Diet and chemical defenses of the Sonoran Desert toad
Source: PLoS One. 2025 Nov 10;20(11):e0335661. doi: 10.1371/journal.pone.0335661 (PMC12599970; doi:10.1371/journal.pone.0335661)
Supplement: S1 Text — (DOCX) [file pone.0335661.s006.docx]

**S1 Text. Additional Description of Diet Data for Sympatric Anurans**

As in Sonoran Desert toads, hymenopterans and coleopterans (beetles) were the dominant prey items, with 37 ants (57.8%) in the stomach contents of 9 anurans, and 19 beetles (29.7%) in the stomach contents of 7 anurans (for species-specific prey type breakdowns, see S4 Table). We encountered 2 introduced black webspinners (*Oligotoma nigra*) in the stomach of a Couch’s spadefoot from urban habitat; all other prey items identified were native species. We found plant matter in the stomach contents of at least one individual of all three species, and rocks or sand in the stomach contents of one Great Plains toad and one Couch’s spadefoot.
