# Supplementary material for: Diet and chemical defenses of the Sonoran Desert toad
Source: PLoS One. 2025 Nov 10;20(11):e0335661. doi: 10.1371/journal.pone.0335661 (PMC12599970; doi:10.1371/journal.pone.0335661)
Supplement: Attachment — Submitted filename: Luccioni et al. 2025_PLOS One_Response to Reviewers.docx [file pone.0335661.s008.docx]

August 28, 2025

Editorial Board of PLOS One

Response to Review Comments for Manuscript PONE-D-24-26523: Diet and chemical defenses of the Sonoran Desert toads

*************************************************************************************************************

***Response:*** *Thank you very much for the detailed review of our manuscript and for the opportunity to make revisions. We are grateful for your feedback and that of the two anonymous referees. Our revised manuscript was edited accordingly. Below we respond in detail to all comments from yourself and both reviewers in italics with review comments provided in normal font for your reference.*

When submitting your revision, we need you to address these additional requirements.

1. Please ensure that your manuscript meets PLOS ONE's style requirements, including those for file naming. The PLOS ONE style templates can be found at

<https://journals.plos.org/plosone/s/file?id=wjVg/PLOSOne_formatting_sample_main_body.pdf> and

[https://journals.plos.org/plosone/s/file?id=ba62/PLOSOne_formatting_sample_title_authors_affiliations.pdf](https://track.editorialmanager.com/CL0/https:%2F%2Fjournals.plos.org%2Fplosone%2Fs%2Ffile%3Fid=ba62%2FPLOSOne_formatting_sample_title_authors_affiliations.pdf/1/010f01961b448d36-7b1c080a-20c1-4266-acb0-f931a1f8edcf-000000/f4OAau8wHVgVkLnV9yTxJbzeMSeK8tgvNVTzoZ82rY0=206)

***Response:*** *We appreciate your providing these templates, and have now ensured that our manuscript meets all style requirements of the journal.*

2. In your Methods section, please provide additional information regarding the permits you obtained for the work. Please ensure you have included the full name of the authority that approved the field site access and, if no permits were required, a brief statement explaining why.

***Response:*** *All work was covered under Arizona Game and Fish Department (AZGFD) scientific collecting licenses. All field sites were on public land, approved by AZGFD for sampling, and specifically covered under our licenses. We have updated our Methods section to clarify this (see lines 136-139).*

3. To comply with PLOS ONE submissions requirements, in your Methods section, please provide additional information regarding the experiments involving animals and ensure you have included details on (1) methods of sacrifice, (2) methods of anesthesia and/or analgesia, and (3) efforts to alleviate suffering.

***Response:*** *The requested additional information for (1-3) is now provided in lines 139-143.*

4. Thank you for stating the following in the Acknowledgments Section of your manuscript:
“We are grateful to a wonderful team who made this study possible: Leisy and Michael Wyman allowed us facilities access and indispensable fieldwork support, Robert Villa shared his expertise regarding wide-ranging aspects of the study and provided a useful review of our manuscript, John Weins at the University of Arizona stored our samples from August to September 2020, Nora Martin advised our stomach flushing protocol, Dave Ramirez provided valuable logistical support and Katie Fiocca shared helpful comments on our manuscript. Many people generously assisted with visual identification of invertebrates from stomach and fecal samples, including Brad Barnd (BugGuide), Michael Bogan, David Donoso, Curt Harden (BugGuide), Solomon Hendrix (BugGuide), Andrew Johnston, Blaine Mathison (BugGuide) and Wyatt Mendez. We also thank Alberto Arevalo and Elizabeth Navarro for their support in creating photographs for select figures, and Quinn Agnew, Daniel Carhuff, Andrew Connoy and Tasman Rosenfeld for their assistance with our fieldwork. This work was funded by a New York Stem Cell Foundation to LAO. LAO is a New York Stem Cell Foundation – Robertson Investigator.”

We note that you have provided funding information that is currently declared in your Funding Statement. However, funding information should not appear in the Acknowledgments section or other areas of your manuscript. We will only publish funding information present in the Funding Statement section of the online submission form.
Please remove any funding-related text from the manuscript and let us know how you would like to update your Funding Statement. Currently, your Funding Statement reads as follows:
“LAO received funding from the New York Stem Cell Foundation (<https://nyscf.org/>) for this work. LAO is a New York Stem Cell Foundation – Robertson Investigator. The New York Stem Cell Foundation did not play any role in the study design, data collection and analysis, decision to publish, or preparation of this manuscript.”

Please include your amended statements within your cover letter; we will change the online submission form on your behalf.

***Response:*** *Thank you for bringing this to our attention. We have removed the following line from our Acknowledgements: “This work was funded by a New York Stem Cell Foundation to LAO. LAO is a New York Stem Cell Foundation – Robertson Investigator.” We would like to maintain the Funding Statement in its current form.*

5. When completing the data availability statement of the submission form, you indicated that you will make your data available on acceptance. We strongly recommend all authors decide on a data sharing plan before acceptance, as the process can be lengthy and hold up publication timelines. Please note that, though access restrictions are acceptable now, your entire data will need to be made freely accessible if your manuscript is accepted for publication. This policy applies to all data except where public deposition would breach compliance with the protocol approved by your research ethics board. If you are unable to adhere to our open data policy, please kindly revise your statement to explain your reasoning and we will seek the editor's input on an exemption. Please be assured that, once you have provided your new statement, the assessment of your exemption will not hold up the peer review process.

***Response:*** *All data are now publicly available through Dryad and Genbank. Accession information is included in the updated version of our manuscript.*

6. We note that Figures 1, 2, 3, 4, 5 and S1 in your submission contain copyrighted images. All PLOS content is published under the Creative Commons Attribution License (CC BY 4.0), which means that the manuscript, images, and Supporting Information files will be freely available online, and any third party is permitted to access, download, copy, distribute, and use these materials in any way, even commercially, with proper attribution. For more information, see our copyright guidelines: <http://journals.plos.org/plosone/s/licenses-and-copyright>.

We require you to either (1) present written permission from the copyright holder to publish these figures specifically under the CC BY 4.0 license, or (2) remove the figures from your submission:

a. You may seek permission from the original copyright holder of Figures 1, 2, 3, 4, 5 and S1 to publish the content specifically under the CC BY 4.0 license.

We recommend that you contact the original copyright holder with the Content Permission Form (<http://journals.plos.org/plosone/s/file?id=7c09/content-permission-form.pdf>) and the following text:
“I request permission for the open-access journal PLOS ONE to publish XXX under the Creative Commons Attribution License (CCAL) CC BY 4.0 (<http://creativecommons.org/licenses/by/4.0/>). Please be aware that this license allows unrestricted use and distribution, even commercially, by third parties. Please reply and provide explicit written permission to publish XXX under a CC BY license and complete the attached form.”

Please upload the completed Content Permission Form or other proof of granted permissions as an "Other" file with your submission.

In the figure caption of the copyrighted figure, please include the following text: “Reprinted from [ref] under a CC BY license, with permission from [name of publisher], original copyright [original copyright year].”

b. If you are unable to obtain permission from the original copyright holder to publish these figures under the CC BY 4.0 license or if the copyright holder’s requirements are incompatible with the CC BY 4.0 license, please either i) remove the figure or ii) supply a replacement figure that complies with the CC BY 4.0 license. Please check copyright information on all replacement figures and update the figure caption with source information. If applicable, please specify in the figure caption text when a figure is similar but not identical to the original image and is therefore for illustrative purposes only.

***Response:*** *All photographs included in the manuscript are the property of the authors.*

7. [We note that Figure 2 in your submission contain map/satellit] image which may be copyrighted. All PLOS content is published under the Creative Commons Attribution License (CC BY 4.0), which means that the manuscript, images, and Supporting Information files will be freely available online, and any third party is permitted to access, download, copy, distribute, and use these materials in any way, even commercially, with proper attribution. For these reasons, we cannot publish previously copyrighted maps or satellite images created using proprietary data, such as Google software (Google Maps, Street View, and Earth). For more information, see our copyright guidelines: http://journals.plos.org/plosone/s/licenses-and-copyright.

We require you to either (1) present written permission from the copyright holder to publish these figures specifically under the CC BY 4.0 license, or (2) remove the figures from your submission:

a. You may seek permission from the original copyright holder of Figure 2 to publish the content specifically under the CC BY 4.0 license.

We recommend that you contact the original copyright holder with the Content Permission Form (http://journals.plos.org/plosone/s/file?id=7c09/content-permission-form.pdf) and the following text:
“I request permission for the open-access journal PLOS ONE to publish XXX under the Creative Commons Attribution License (CCAL) CC BY 4.0 (http://creativecommons.org/licenses/by/4.0/). Please be aware that this license allows unrestricted use and distribution, even commercially, by third parties. Please reply and provide explicit written permission to publish XXX under a CC BY license and complete the attached form.”

Please upload the completed Content Permission Form or other proof of granted permissions as an "Other" file with your submission.

In the figure caption of the copyrighted figure, please include the following text: “Reprinted from [ref] under a CC BY license, with permission from [name of publisher], original copyright [original copyright year].”

b. If you are unable to obtain permission from the original copyright holder to publish these figures under the CC BY 4.0 license or if the copyright holder’s requirements are incompatible with the CC BY 4.0 license, please either i) remove the figure or ii) supply a replacement figure that complies with the CC BY 4.0 license. Please check copyright information on all replacement figures and update the figure caption with source information. If applicable, please specify in the figure caption text when a figure is similar but not identical to the original image and is therefore for illustrative purposes only.
The following resources for replacing copyrighted map figures may be helpful:

USGS National Map Viewer (public domain): http://viewer.nationalmap.gov/viewer/
The Gateway to Astronaut Photography of Earth (public domain): http://eol.jsc.nasa.gov/sseop/clickmap/
Maps at the CIA (public domain): https://www.cia.gov/library/publications/the-world-factbook/index.html and https://www.cia.gov/library/publications/cia-maps-publications/index.html
NASA Earth Observatory (public domain): http://earthobservatory.nasa.gov/
Landsat: http://landsat.visibleearth.nasa.gov/
USGS EROS (Earth Resources Observatory and Science (EROS) Center) (public domain): http://eros.usgs.gov/#
Natural Earth (public domain):](http://track.editorialmanager.com/CL0/We%2520note%2520that%2520Figure%25202%2520in%2520your%2520submission%2520contain%2520map%2Fsatellit%255d%2520image%2520which%2520may%2520be%2520copyrighted.%2520All%2520PLOS%2520content%2520is%2520published%2520under%2520the%2520Creative%2520Commons%2520Attribution%2520License%2520(CC%2520BY%25204.0),%2520which%2520means%2520that%2520the%2520manuscript,%2520images,%2520and%2520Supporting%2520Information%2520files%2520will%2520be%2520freely%2520available%2520online,%2520and%2520any%2520third%2520party%2520is%2520permitted%2520to%2520access,%2520download,%2520copy,%2520distribute,%2520and%2520use%2520these%2520materials%2520in%2520any%2520way,%2520even%2520commercially,%2520with%2520proper%2520attribution.%2520For%2520these%2520reasons,%2520we%2520cannot%2520publish%2520previously%2520copyrighted%2520maps%2520or%2520satellite%2520images%2520created%2520using%2520proprietary%2520data,%2520such%2520as%2520Google%2520software%2520(Google%2520Maps,%2520Street%2520View,%2520and%2520Earth).%2520For%2520more%2520information,%2520see%2520our%2520copyright%2520guidelines:%2520http:%2Fjournals.plos.org%2Fplosone%2Fs%2Flicenses-and-copyright.%250b%250bWe%2520require%2520you%2520to%2520either%2520(1)%2520present%2520written%2520permission%2520from%2520the%2520copyright%2520holder%2520to%2520publish%2520these%2520figures%2520specifically%2520under%2520the%2520CC%2520BY%25204.0%2520license,%2520or%2520(2)%2520remove%2520the%2520figures%2520from%2520your%2520submission:%250b%250ba.%2520You%2520may%2520seek%2520permission%2520from%2520the%2520original%2520copyright%2520holder%2520of%2520Figure%25202%2520to%2520publish%2520the%2520content%2520specifically%2520under%2520the%2520CC%2520BY%25204.0%2520license.%2520%250b%250bWe%2520recommend%2520that%2520you%2520contact%2520the%2520original%2520copyright%2520holder%2520with%2520the%2520Content%2520Permission%2520Form%2520(http:%2Fjournals.plos.org%2Fplosone%2Fs%2Ffile%3Fid=7c09%2Fcontent-permission-form.pdf)%2520and%2520the%2520following%2520text:%250b%E2%80%9CI%2520request%2520permission%2520for%2520the%2520open-access%2520journal%2520PLOS%2520ONE%2520to%2520publish%2520XXX%2520under%2520the%2520Creative%2520Commons%2520Attribution%2520License%2520(CCAL)%2520CC%2520BY%25204.0%2520(http:%2F%2Fcreativecommons.org%2Flicenses%2Fby%2F4.0%2F).%2520Please%2520be%2520aware%2520that%2520this%2520license%2520allows%2520unrestricted%2520use%2520and%2520distribution,%2520even%2520commercially,%2520by%2520third%2520parties.%2520Please%2520reply%2520and%2520provide%2520explicit%2520written%2520permission%2520to%2520publish%2520XXX%2520under%2520a%2520CC%2520BY%2520license%2520and%2520complete%2520the%2520attached%2520form.%E2%80%9D%250b%250bPlease%2520upload%2520the%2520completed%2520Content%2520Permission%2520Form%2520or%2520other%2520proof%2520of%2520granted%2520permissions%2520as%2520an%2520%2522Other%2522%2520file%2520with%2520your%2520submission.%250b%250bIn%2520the%2520figure%2520caption%2520of%2520the%2520copyrighted%2520figure,%2520please%2520include%2520the%2520following%2520text:%2520%E2%80%9CReprinted%2520from%2520%255bref%255d%2520under%2520a%2520CC%2520BY%2520license,%2520with%2520permission%2520from%2520%255bname%2520of%2520publisher%255d,%2520original%2520copyright%2520%255boriginal%2520copyright%2520year%255d.%E2%80%9D%250b%250bb.%2520If%2520you%2520are%2520unable%2520to%2520obtain%2520permission%2520from%2520the%2520original%2520copyright%2520holder%2520to%2520publish%2520these%2520figures%2520under%2520the%2520CC%2520BY%25204.0%2520license%2520or%2520if%2520the%2520copyright%2520holder%E2%80%99s%2520requirements%2520are%2520incompatible%2520with%2520the%2520CC%2520BY%25204.0%2520license,%2520please%2520either%2520i)%2520remove%2520the%2520figure%2520or%2520ii)%2520supply%2520a%2520replacement%2520figure%2520that%2520complies%2520with%2520the%2520CC%2520BY%25204.0%2520license.%2520Please%2520check%2520copyright%2520information%2520on%2520all%2520replacement%2520figures%2520and%2520update%2520the%2520figure%2520caption%2520with%2520source%2520information.%2520If%2520applicable,%2520please%2520specify%2520in%2520the%2520figure%2520caption%2520text%2520when%2520a%2520figure%2520is%2520similar%2520but%2520not%2520identical%2520to%2520the%2520original%2520image%2520and%2520is%2520therefore%2520for%2520illustrative%2520purposes%2520only.%250bThe%2520following%2520resources%2520for%2520replacing%2520copyrighted%2520map%2520figures%2520may%2520be%2520helpful:%250b%250bUSGS%2520National%2520Map%2520Viewer%2520(public%2520domain):%2520http:%2F%2Fviewer.nationalmap.gov%2Fviewer%2F%250bThe%2520Gateway%2520to%2520Astronaut%2520Photography%2520of%2520Earth%2520(public%2520domain):%2520http:%2F%2Feol.jsc.nasa.gov%2Fsseop%2Fclickmap%2F%250bMaps%2520at%2520the%2520CIA%2520(public%2520domain):%2520https:%2F%2Fwww.cia.gov%2Flibrary%2Fpublications%2Fthe-world-factbook%2Findex.html%2520and%2520https:%2F%2Fwww.cia.gov%2Flibrary%2Fpublications%2Fcia-maps-publications%2Findex.html%250bNASA%2520Earth%2520Observatory%2520(public%2520domain):%2520http:%2F%2Fearthobservatory.nasa.gov%2F%250bLandsat:%2520http:%2F%2Flandsat.visibleearth.nasa.gov%2F%250bUSGS%2520EROS%2520(Earth%2520Resources%2520Observatory%2520and%2520Science%2520(EROS)%2520Center)%2520(public%2520domain):%2520http:%2F%2Feros.usgs.gov%2F%23%2520Natural%2520Earth%2520(public%2520domain):%2520http:%2F%2Fwww.naturalearthdata.com%2F/1/010f01961b448d36-7b1c080a-20c1-4266-acb0-f931a1f8edcf-000000/b1J7_mhONG6SVEzT4Oleg8xTSOzc9JLH_P7J4SSdAiY=206) <http://www.naturalearthdata.com/>

***Response:*** *Confirmation of a CC-BY license has been obtained for the map from Figure 2 which was obtained from publicly available GIS Open Data Pima County Maps (*[*https://gisopendata.pima.gov/*](https://gisopendata.pima.gov/)*).*

8. Please include captions for your Supporting Information files at the end of your manuscript, and update any in-text citations to match accordingly. Please see our Supporting Information guidelines for more information: http://journals.plos.org/plosone/s/supporting-information.

***Response:*** *Captions are now provided.*

9. Please review your reference list to ensure that it is complete and correct. If you have cited papers that have been retracted, please include the rationale for doing so in the manuscript text, or remove these references and replace them with relevant current references. Any changes to the reference list should be mentioned in the rebuttal letter that accompanies your revised manuscript. If you need to cite a retracted article, indicate the article’s retracted status in the References list and also include a citation and full reference for the retraction notice.

***Response:*** *We have reviewed our references list and ensured it is complete and correct. Changes from the original submission are as follows:*

1) *We rectified our citation of [33] (Brown, DE. Biotic communities: southwestern United States and northwestern Mexico. University of Utah Press; 1994.), which was originally cited in the text but not included in the References list.*
2) *We added a reference to our Materials and methods section [48] to clarify our data visualization methods.*
3) *We added two references to our Discussion section, [61] and [62]. These articles represent new findings since the conclusion of our study that are relevant to the interpretation of our results.*
4) *Where necessary, we made minor formatting corrections (e.g. renumbering, removing extra spacing) to our References section and in-text citations.*

Additional Editor Comments:

Dear Dr. Wyman,

Firstly, my apologies for the long delay to reach a decision regarding your manuscript. This delay was due to changes of editor and issues to find available reviewers. To our knowledge, your work represents the first to test whether the toad I. alvarius 5-MeO-DMT secretion is related to a dietary source, together with an interesting diet study.

I have received the reviewer's comments on your manuscript. Based on their evaluation your manuscript is considered accepted upon minor revision. Both reviewers believe that your manuscript is nice and well written and I agree. Although not completely clear due some kind of issue in the Plos platform (please see above), the Reviewer 1 seems to believe that diet is not probably the most likely the answer to the uniqueness of I. alvarius 5-MeO-DMT presence, but more a hypothesis based on bacterial symbiosis or a hydroxylase presence in the toad skin. The reviewer 2 have a similar opinion regarding the origin of 5-MeO-DMT in I. alvarius still not clarified. Perhaps the manuscript could be focus more on testing the hypothesis that the origin of 5-MeO-DMT in I. alvarius is not related do dietary origin rather than testing that the hypothesis that is related. Is a fine line, but it changes the manuscript presentation. Please let me know your thoughts regarding this point.

Please see remaining comments and corrections of the reviewers below. I am looking forward to receive a revised version of your manuscript including replies to the essential critiques and comments of the reviewers through the online editorial system. For those to which you disagree a justification must be presented.  Thank you for submitting your work to PlosOne!   If you have any further questions please let me know.   Natan Maciel.

***Response:*** *We are thrilled to hear of our acceptance pending minor revision. Thank you very much for your compliments about the manuscript, and all of your work finding reviewers and synthesizing and providing feedback. We have done our best to address all comments and concerns herein.*

*In regard to changing the presentation of our hypothesis, while we understand there is good reason to think that 5-MeO-DMT is produced endogenously by an enzyme, we feel that maintaining our hypothesis in its current form is the most honest presentation of our initial aim–investigating diet as a source for 5-MeO-DMT production. Based on the criteria identified by Savitsky et al. [26], outlined in our Introduction,* I. alvarius *is a candidate taxon for defensive sequestration, and we felt our hypothesis was worthy of investigation. We made edits to lines 68-69 in the Introduction and 374-387 in the Discussion, to better explain the evidence and our reasoning regarding this alternate possibility, and clarify the current state of the literature and evidence suggesting endogenous production (which we agree is the most likely option in light of our results).*

*One other point we would like to raise–authors Marina Luccioni (ML) and Jules Wyman (JW) are joint first authors, with equal contributions to the study. In our submission the order of authors specifies JW first with ML second. However, for the publication we would like Marina Luccioni listed first and Jules Wyman second (alphabetical order), with both authors listed as equal contributors and corresponding authors. This change is reflected in our cover page.*

*Thank you for your consideration,*

*Jules and Marina, on behalf of all the authors*

Reviewer 1

This is a well written manuscript that reports the dietary regime of desert toads in an effort to determine if a potent compound 5-Me0--DMT has a dietary source. This reviewer would be more comfortable if there were less effort to sell the idea that these toads are special and

***Response:*** *We thank Reviewer 1 for seeing the merit in our work and for their thoughtful suggestions. Unfortunately, as the comment seems to be cut off, we are not completely clear on what this reviewer means about de-emphasizing that these toads are special. However, we have done our best to amend the manuscript based on the editor’s interpretation of their overall feedback and the reviewer’s specific comments (see above and below), which we hope addresses this reviewer’s concern.*


Abstract
Fine
Intro—Why is work on what used to be called Bufo marinas and other bufonids that have compounds related 5-Me0-DMT not included?

***Response:*** *We have added this literature to the manuscript. See lines 58-60 and 384-387.*

85-86 delete. Does not add to story.

***Response:*** *We respectfully disagree, as we feel that it is important to note that no similar studies have been conducted previously, both as part of a comprehensive review of the existing literature relevant to our study and to clarify our contribution to the field. However, we did remove the discussion of other anurans in these lines, as we agree that this is superfluous and distracts from the main contribution of our research.*

Methods
122- why weren’t samples “suitable”?

***Response:*** *We have clarified this point: “six samples were incorrectly stored, rendering them unsuitable for visual or molecular identification”*

Results
Intro and Discussion
So three additional species contain 5-Me0-tryptamine or a close relative?
That leaves the spadefoot for diet comparison?

***Response:*** *While the two additional bufonid species we sampled produce other related tryptamines,* I. alvarius *is unique in its production of the especially potent 5-MeO-DMT. Our study specifically tested whether 5-MeO-DMT was directly sequestered from a unique dietary source, without comment on the potential source of the other tryptamines, and we therefore compared the diet of* I. alvarius *with the other three, non-5-MeO-DMT-producing species. While we understand the concern this reviewer is raising, we are not able to make statements about whether this class of compounds more generally are obtained from a specialized diet with our current dataset, as we sampled too few spadefoots (n=10, but only 4 with identifiable stomach contents) to accurately compare their diet to that of bufonids. Our preliminary results suggest all four species are generalists with similar diets, but this represents an excellent opportunity for future study!*

Why not look for the hydroxylase?

***Response:*** *Unfortunately, we were not able to attempt to isolate the hypothesized o-methyl-transferase from* I. alvarius *skin. This was due to both pandemic-induced laboratory constraints and that our permits did not include tissue collection. However, we now suggest it as a key next step in untangling this puzzle (see lines 385-387).*

Reviewer 2

The study aims to investigate the origin of the compound 5-MeO-DMT, present in high levels in the skin of the Sonoran toad Incilius alvarius. Although the existence of this compound in this species has been known for a long time, its origin remains unknown. The authors used a comparative ecological, biochemical and molecular biology approach, analyzing the skin secretions and the diet of I. alvarius and three other sympatric toad species from 5 locations with distinctive ecological characteristics. The analysis of this data set showed that the prey consumed by all species is very similar, and all lack 5-MeO-DMT. Thus, the explanation for the existence of the compound solely in I. alvarius must be based on endogenous production or originating from the bacterial flora of the skin of these animals, as was recently demonstrated in the salamander Taricha granulosa, whose skin is very rich in the toxin TTX. The work is well written, well-illustrated, and methodologically appropriate, and it frankly discusses the points that still need further clarification. However, it sheds light on an open question that may pave the way for new investigations capable of better clarifying the actual origin of 5-MeO-DMT in I. alvarius.

***Response:*** *We thank Reviewer 2 very much for providing a summary of the manuscript, which aligns with the message we hoped to convey, and for their helpful comments and corrections. We address all specific feedback below.*

Below are a few comments on points I observed while reading the text.

Line 47 – Taricha granulosa??

***Response:*** *Thank you for pointing out this error–we have corrected it.*

Line 57 – I believe “agitated” is not the best term here. Maybe “theatened” or “attacked”??

***Response:*** *We agree with the reviewer’s suggestion and have changed the term in question to “threatened.”*

Table 1, legend - Sc = Scaphiopus couchii (and not Sceloporus couchii)

*We have corrected this error.*

Line 132 – “Secretions were collected in the field using kimwipes”. I understand that the authors are referring here to the secretion coming from the parotoids. Did the animals naturally release the skin secretion, or were they mechanically stimulated to secrete? This point could be better explained.

***Response:*** *We clarified that the stimulation was manual (but gentle).*

*We made other minor changes to the manuscript.*

*First, in light of recent published and anecdotal evidence of heavy collecting pressure on* I. alvarius *to harvest their 5-MeO-DMT for recreational use (see lines 103-105), we have chosen to remove information about the exact locations at which we sampled, and instead describe the general areas and habitats. This affects Table 1 (removed coordinates) and Figure 2 (noted that plotted locations are approximate). Coordinates can be obtained from corresponding authors upon request.*

*Second, we added comments on two recent studies ([61], [62]) to the Discussion. These lend support to the hypothesis of endogenous production/biosynthesis of 5-MeO-DMT, but were not published at the time we conducted our research.*

*Third, we have standardized the naming of all study species throughout the manuscript, referring to them by abbreviated scientific name after the first mention of the complete scientific name.*

*Fourth, we corrected an error that we noticed in figures 4 and 5–we edited the proportions of variance explained by each principal component to reflect the correct values. We had accidentally retained values from a preliminary analysis rather than the final analysis of the full dataset. These updates did not affect any results or interpretations.*

*Fifth, due to the time elapsed since initial analyses, we re-analyzed all barcode sequences using the current version of Geneious Prime (2025.1.3) and up-to-date NCBI Genbank nr database. These updates produced minimal changes to results, and did not affect interpretations.*

*Sixth, we have minorly amended descriptions of our chemical sampling protocol and included additional details within Tables S2 and S3 for clarity. These updates did not affect our results or interpretations.*

*Finally, we have made slight edits to our wording to improve clarity and concision, all of which are noted in the revised version of the manuscript.*

*Many thanks for your consideration.*
